# Post-natal prognostic factors in CDH: experience of 11 years in a referral center in Brazil

**DOI:** 10.1016/j.clinsp.2023.100217

**Published:** 2023-05-27

**Authors:** Camila Pinho Brasileiro Martins Nam, Carolina Vieira Campos, Gabriela Nunes Leal, Uenis Tannuri, Maria Esther Jurfest Rivero Ceccon, Werther Brunow de Carvalho

**Affiliations:** a Pediatric Department, Instituto da Criança do Hospital das Clínicas da Faculdade de Medicina da Universidade de São Paulo, São Paulo, SP, Brazil; b Pediatric Cardiology Department, Instituto do Coração do Hospital das Clínicas da Faculdade de Medicina da Universidade de São Paulo, São Paulo, SP, Brazil; c Echocardiography Laboratory of Instituto da Criança do Hospital das Clínicas da Faculdade de Medicina da Universidade de São Paulo, São Paulo, SP, Brazil

**Keywords:** Congenital Diaphragmatic Hernia, Neonatal Intensive Care, Oxygenation Index, Mortality, Prognosis

## Abstract

**Objective:**

To describe post-natal risk factors associated with death in Newborns (NB) with Congenital Diaphragmatic Hernia (CDH) in a Brazilian reference center.

**Methods:**

In this retrospective cohort study, post-natal clinical factors of all NB diagnosed with CDH were reviewed in an 11-year period (2007‒2018). The primary outcome was death. Secondary outcomes included clinical features, prognostic indexes, type of mechanical ventilation, complications during hospitalization and surgical repair.

**Results:**

After applying the exclusion criteria, the authors analyzed 137 charts. Overall mortality was 59% (81/137), and the highest rates were observed for low-birth-weight NB (87%), syndromic phenotype (92%), and those with major malformations (100%). Prognostic indexes such as Apgar, SNAPPE-II and 24hOI (best oxygenation index in 24 hours) were all associated with poor evolution. In a multivariate analysis, only birth weight and 24hOI were statistically significant risk factors for mortality, with a reduction in mortality risk of 17.1% (OR = 0.829, 95% IC 0.72‒0.955, p = 0.009) for each additional 100g at birth and an increase by 26.5% (OR = 1.265, 95% IC 1.113‒1.436, p = 0.0003) for each unitary increase at the 24hOI.

**Conclusion:**

Prognostic indexes are an important tool for predicting outcomes and improving resource allocation. Post-natal risk factors may be more suitable for settings where antenatal diagnosis is not universal. Classical risk factors, such as prematurity, low birth weight, higher need for supportive care, and poorer prognostic indexes were associated with mortality in our CDH population.

## Highlights

• Clinical features, prognostic indexes and echocardiographic parameters may contribute together to better define survival chances in CDH.
• When antenatal factors are not available, postnatal factors can be good predicting tools and may suffice to assess the prognosis of CDH patients.

## Introduction

Although there have been several therapeutic advances over the last decades, Congenital Diaphragmatic Hernia (CDH) is still a disease with high mortality, ranging from 30% to 70% in recently published studies.^1^^,^^2^ The myriad of phenotypic presentations and the variety of both heart and lung involvement cause spectral outcomes. Antenatal parameters obtained in fetal echocardiogram, magnetic resonance, and ultrasound, as the observed and expected Lung-to-Head ratio (LHR o/e) may offer some parameters that can help to infer prognosis.^3^^,^^4^ This index may be a guide to consider Fetoscopic Tracheal Occlusion (FETO), a procedure that has been shown to improve survival.^5^

The pathophysiological basis for this disease includes Pulmonary Hypertension (PH) and cardiac dysfunction.^6^^,^^7^ Association with risk factors such as intrathoracic liver, right-sided hernia defect, low birth weight, prematurity, genetic syndromes, and other congenital abnormalities may drastically worsen the chances of survival.^8^

Unfortunately, the reality of a heterogeneous antenatal diagnosis and follow-up, associated with the illegality of pregnancy interruption observed in many developing countries may contribute to a scenario of worse outcomes.^9^ Identifying post-natal risk factors for mortality may help to arrange optimal goals of care for this critical population. The authors carried out the present study with the aim of describing and analyzing postnatal factors related to the grim prognosis of these Newborns (NB). The main goal was to define risk factors associated with death in newborns with CDH who were admitted to our service during the study period.

## Materials and methods

### Study population

The authors performed a retrospective cohort study based on a chart review of medical records from Newborns (NB) diagnosed with CDH admitted over an 11-year period (2007‒2018) at the University of Sao Paulo – School of Medicine teaching hospital's Neonatal Intensive Care Unit (NICU), a tertiary service and national reference center for CDH, where Extracorporeal Membrane Oxygenation (ECMO) is not available routinely. Consent was waived due to the retrospective nature of the study. The Institutional Ethics Committee on Human Research approved the study protocol.

NB with CDH characterized by a diaphragmatic defect who were treated at our Institution were included in the study. NB with diaphragmatic eventration, Cantrell's Pentalogy, Siamese twins and those who had surgical correction at another hospital were excluded from the analysis.

### Clinical data

Data on antenatal diagnosis, maternal, delivery, gender, Gestational Age (GA), birth weight, FETO procedure, intrathoracic liver, hernia laterality, associated extra-cardiac MF, neonatal scores (Apgar, SNAPPE-II – Score for Neonatal Acute Physiology with Perinatal Extension-II, best Oxygenation Index (OI) in 24 hours of life – 24hOI*), clinical outcomes (High-Frequency Oscillatory Ventilation – HFOV and Vasoactive Drugs – VAD use), complications during hospitalization (sepsis, pneumothorax, coagulation disorders, cardiac arrest, seizure or acute kidney injury), surgical repair and requirement of the prosthetic patch were recorded for each neonate. The primary outcome was death.

* 24hOI was calculated according to the formula: FiO_2_ × MAP × 100/PaO_2_, where FiO_2_ indicates the fraction of inspired oxygen, MAP mean airway pressure, and PaO_2_ partial pressure of oxygen in arterial blood. PaO_2_ was obtained according to gasometrical analysis of arterial blood, and FiO_2_ and MAP according to ventilatory parameters at the time of blood sample collection.

Post-natal echocardiographic parameters were also analyzed (Tables 1 and 2) and a comparison between non-survivors and survivors was performed for those NB without congenital heart anomalies who had an exam performed over the first 72 hours of life.

**Table 1:** Echocardiographic parameters evaluated in M-mode and Doppler study.

**1) M-Mode, parasternal short axis view:**
a) Right ventricle end-diastolic diameter (RV EDD)
b) Left ventricle end-diastolic diameter (LV EDD)
c) Left ventricle end-systolic diameter (LV ESD)
d) Left ventricle ejection fraction (LVEF), calculated by Teichholz^13^ method.
e) Aortic root diameter (Ao)
g) Tricuspid Annular Plane Systolic Excursion (TAPSE)
**2) Doppler Exam:**
a) Systolic pulmonary artery pressure (SPAP) estimated via tricuspid insufficiency (TI).
b) Ductal shunt flow direction
c) Presence of midsystolic notch in pulmonary artery Doppler curve
d) Velocity-Time Integral calculation, pulmonary (VTIp)
**Relevant information:**
Z-score values of the diameters obtained by the M-Mode were calculated using as reference the publication by Kampmann et al., 2000.^10^
TI was calculated according to the American Society of Echocardiography Guideline.^11^
SPAP obtained through TI (tricuspid insufficiency) was compared to the non-invasive systemic Systolic Blood Pressure (SBP) value recorded in time of the examination, allowing the identification of patients with SPAP ≥ SBP. In the absence of TI, SPAP ≥ SBP was identified when the duct shunt flow was bidirectional or when there was a predominance of flow directed from the pulmonary artery to the aorta. In the absence or TI or ductus arteriosus, SPAP ≥ SBP was identified when there was a midsystolic notch in the Doppler curve of pulmonary flow.^12^

**Table 2:** Classifications of *Ductal shunt* and of the relation between SPAP and SBP.

Ductal shunt	Classification
Exclusively Ao-PA	1
Bidirectional, predominantely Ao-PA	2
Bidirectional, predominantely Ao-PA or exclusively Ao-PA, midsystolic notch	3

Relation SPAP/SBP	Classification
SPAP < 2/3 of SBP or ductal shunt type 1	1
SPAP ≥ 2/3 of SBP or ductal shunt type 2	2
SPAP ≥ SBP or ductal shunt type 3	3

Ao, Aorta; PA, Pulmonary Artery; SPAP, Systolic Pressure of the Pulmonary Artery; SBP, Systemic Arterial Pressure.

### Statistical analysis

Descriptive statistical analysis was performed in order to describe the quantitative variables, using measures of central tendency (mean and median) and variability (minimum, maximum and standard deviation). Qualitative variables were presented by absolute frequencies (n) and percentages (%). Student's *t-*test was used to compare the measures of numerical variables that had a normal distribution; in the case of asymmetric distribution, the Mann-Whitney test was used. When two or more groups were compared, Kruskal-Wallis non-parametric test was used. To assess the association between two qualitative variables, Pearson's Chi-Square test or Fisher's exact test were considered, when appropriate. The correlation between two quantitative variables was measured by Pearson or Spearman´s correlation coefficient, depending on the variables' characteristics.

Patients were divided into two groups according to the evolution (Survivors vs non-survivors). Statistical analysis based on previously mentioned tests was performed to compare both groups. In order to measure the effect size on the strength of association between assessed variables the authors used the Odds Ratio (OR) with a Confidence Interval 95% (95% CI). The significance level adopted was 5%. Multiple logistic regression analysis was conducted successively on factors that had been significant in the univariate analyses and have had clinical importance. Data analysis was performed using IBM SPSS version 20 software.

## Results

**Fig. 1:**
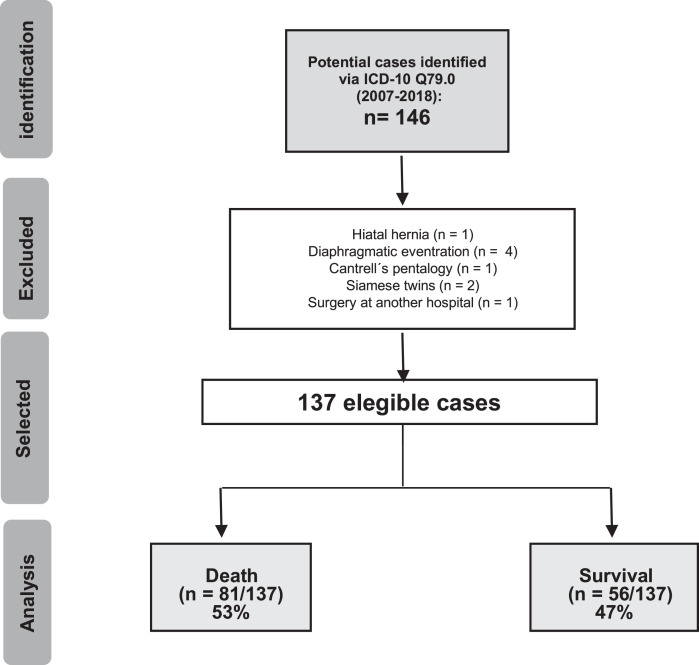
Flowchart of included patients. Total number of patients who died: *n* = 81/137 (53.1%).

The authors identified 146 NB with the diagnosis of CDH in the study period. After applying the exclusion criteria, the authors analyzed 137 charts (Fig. 1). NB characteristics are described in Table 3. After delivery and neonatal resuscitation, NB was transferred to the NICU where the institutional protocol was followed (supplementary material), including a gentle ventilation strategy, end-organ dysfunction monitoring and other congenital anomalies investigation and treatment. At our center, High-Frequency Oscillatory Ventilation (HFOV) is a rescue modality and ECMO is not an available therapy. Surgical intervention was performed only after cardiopulmonary stability was achieved.

**Table 3:** Patient characteristics.

Variable	n = 137
Gestational age (week)	37.2 ± 2.5
Prematurity, n (%)	44 (32)
Birth weight (g)	2719 ± 618
Low Birth Weight, n (%)	45 (33)
Small for gestational age, n (%)	22 (16)
Apgar 1 min	5 (0‒9)
Apgar 5 min	8 (0‒10)
SNAPPE-II	41 + 20
Fetoscopic endotracheal occlusion, n (%)	28 (20)
Male gender, n (%)	91 (66)
Left-sided defect, n (%)	115 (84)
Liver position: intrathoracic, n (%)	79 (58)
Type of repair, n (%)	
Patch correction	27 (20)
Primary closure	42 (30)
No repair	68 (50)
24hOI	21.5 + 20.3
Treatment with nitric oxide, n (%)	91 (66)
Treatment with inotropes, n (%)	124 (90)
High-frequency oscillation, n (%)	88 (64)
Age at repair (d)	4 (1‒49)

Results are presented as n (%), mean ± SD or median (min‒max).

### Prenatal data

Mean maternal age was 28±6.6 years and 48/137 (35%) of mothers had comorbidities during pregnancy, most frequently premature rupture of membrane and chorioamnionitis 22/137 (18%), hypertension 19/137 (14%) and diabetes 7/137 (5%). Previous diagnoses included obesity 7/137 (5) and asthma 4/137 (3%); illicit drugs were used by 2/137 (1%) and 11/137 (8%) smoked. FETO was performed in 28/137 (20%) patients. CDH diagnosis was performed prenatally in 118/137 (86%) of cases and 104/137 (76%) mothers had pre-natal follow-up at our center. NB delivered at our institution accounted for 118/137 (86%) cases.

### NB characteristics

Most frequently, CDH was left-sided 84% (115/137) and intra-thoracic liver was a feature in 79 cases (58%). Other congenital defects were present in 62/137 (45%) cases, and the most common abnormalities were genitourinary tract anomalies 32/137 (23%) and congenital heart disease 30/137 (22%) (Table 3). The genetic syndrome was suspected clinically in 63/137 (46%) patients, but only 3 had major chromosomal abnormalities: Turner syndrome (45×0), Edwards syndrome (47 XX+18), and another non-specified alteration [46, XX, add (20) (p13)].

Postnatally, all patients required Conventional Mechanical Ventilation (CMV) and 88/137 (64%) also required HFOV. Vasoactive drugs were used in 124/137 patients (90%) and nitric oxide by 91/137 (73%). Surgical correction was possible in 69/137 (50%) patients, and 27/69 (39%) required a patch. The average time until the procedure was 4 days (1‒49 days). At least 69% of the patients had at least one kind of complication, the most frequent one being sepsis (47%), coagulation disorders (34%), and pneumothorax (23%) (Table 3).

### Univariate analysis

Overall mortality was 59% (81/137), and the highest rates were observed for low-birth-weight NB (87%), syndromic phenotype (92%), and those with major malformations (100%). Other NB features associated with mortality were female gender, premature birth, left-sided hernia, and other congenital anomalies. The need for HFOV, inhaled nitric oxide and vasoactive support was also related to worse outcomes. All prognostic indexes were associated with mortality, with cutoff values for SNAPPE-II index of 62 (OR = 1.075 95% CI 1.048‒1.104; p < 0.0001) and 24hOI of 27 (OR = 1.236, 95% CI 1.132‒1.35; p < 0.0001) correlating with 100% chance of death.

Both pneumothorax and coagulation disorders, albeit frequent complications, were not associated with mortality (pneumothorax: 71% vs. 56%, p = 0.127, coagulation disorders 67% vs. 55%, p = 0.162).

Prognostic indexes were significantly associated with mortality: Apgar scores at 1 and 5 minutes were significantly lower for those who did not survive (Apgar 1 min (4 [0‒8] vs. 7 [1‒9], p < 0.0001), Apgar 5 min (7 [0‒9] vs. 9 [4‒10], p < 0.0001). Similarly, non-survivors had worse SNAPPE-II scores, in comparison with those who survived (non-survivors 52 ± 20 vs. survivors 21±18, p < 0.0001). 24hOI was also a relevant mortality risk factor (non-survivors 30.1±20.8 vs. survivors 6.6 ± 5.2, p < 0.0001) (Table 4).

**Table 4:** Risk factors for mortality.

Variable	Death	Survival	p
	n = 81	n = 56	
Prenatal Diagnosis	79 (67)	39 (33)	<0.0001
Prenatal Follow-up at our center	67 (64)	37 (36)	0.025
Birth at another center	3 (16)	16 (84)	0.001
Female Gender	33 (72)	13 (28)	0.033
Prematurity	33 (75)	11 (25)	0.009
Low Birth Weight	39 (87)	6 (13)	<0.0001
Small for gestational age	15 (68)	7 (32)	0.346
Tracheal Balloon	17 (61)	11 (39)	0.848
Left-sided hernia	62 (54)	53 (46)	0.004
Other congenital anomalies	49 (79)	13 (21)	<0.0001
Syndromic phenotype	11 (92)	1 (8)	0.027
Congenital heart disease	25 (83)	5 (17)	0.002
Intra-thoracic liver	53 (67)	26 (33)	0.013
Apgar 1 min	4 (0‒8)	7 (1‒9)	<0.0001
Apgar 5 min	7 (0‒9)	9 (4‒10)	<0.0001
SNAPPE-II	52 ± 20	21 ± 18	<0.0001
Time to surgical intervention (d)	3 (1‒49)	5 (1‒36)	0.926
Need for patch closure	7 (26)	20 (74)	0.228
24hOI	30.1 ± 20.8	6.6 ± 5.2	<0.0001

Results are presented as n (%), mean ± SD or median (min‒max). 24hOI, Best Oxygenation Index during firs 24h of life.

In the subgroup of NB with no heart defects and who had early echocardiogram there was no statistical difference when comparing RV EDD, LV ESD z-scores, and TAPSE. Echocardiographic measurements that were statistically associated with mortality were the LV EDD z-score, LVEF, aortic root and VTIp. Both ductal shunt and SPAP/SBP scales were also relevant risk factors (Table 5).

**Table 5:** Qualitative analysis of the echocardiographic variables assessed.

	Survivors (n = 33); Mean ± SD or Mean (min‒max)	Non-survivors (n = 42); Mean ± SD or Mean (min‒max)	Total Mean ± SD or Mean (min‒max)	N total	p-value
RV EDD z-score	0.87 ± 1.26	0.94 ± 1.34	0.89 ± 1.29	75	0.582
LV EDD z-score	-2.43 ± 1.74	-3.75 ± 1.91	-3.01 ± 1.92	75	0.008
LV ESD z-score	-2.96 ± 1.88	-3.22 ± 3.17	-3.07 ± 2.51	74	0.987
LV EF (%)	78 (29‒93)	64 (20‒95)	74 (20‒95)	75	<0.0001
Aortic root (mm)	8 (6‒11)	7 (4‒11)	8 (4‒11)	73	0.015
TAPSE (cm)	0.685 (0.10‒1.00)	0.675 (0.40‒1.50)	0.68 (0.10‒1.50)	34	0.746
VTIp (cm)	7.8 (4.9‒13.7)	6.62 (3.0‒11.0)	7.5 (3.0‒14.0)	43	0.031
Ductal shunt*					
1	1 (7)	14 (93)	15 (20)		
2	2 (14)	12 (86)	14 (19)	75	<0.0001
3	30 (65)	16 (35)	46 (61)		
SPAP × SBP*					
SPAP < 2/3 SBP	8 (100)	0 (0)	8 (10)		
SPAP ≥ 2/3 SBP	23 (66)	12 (34)	35 (47)	75	0.001
SPAP ≥ SBP	11 (34)	21 (66)	32 (43)		

### Multivariate analysis

After adjusted multivariate analysis, only birth weight and 24hOI were statistically significant risk factors for mortality, with a reduction in mortality risk of 17.1% (OR = 0.829, 95% IC 0.72‒0.955, p = 0.009) for each additional 100g at birth and an increase by 26.5% (OR = 1.265, 95% IC 1.113‒1.436, p = 0.0003) for each unitary increase at the 24hOI (Table 6).

**Table 6:** Multivariate analysis.

Variable	Category	Coefficient	SE	OR	95% CI		p-value
					Inferior	Superior	
Birth weight	100 grams – unity	-0.187	0.072	0.829	0.720	0.955	**0.009**
24hOI	1 – unity	0.235	0.065	1.265	1.113	1.436	**0.0003**
5-minute Apgar score	1 - unity	-0.482	0.264	0.617	0.368	1.035	0.067
Extra-cardiac malformations	No Yes	1.452	0.757	4.270	0.968	18.840	0.055

OR, Odds Ratio; SE, Standard Error; CI, Confidence Interval.

In the subgroup of NB with an early echocardiogram and no heart defects, the authors noticed that for each unit of increase in 24hOI, there was a 15% increase in the chance of death (OR = 1.15; 95% CI 1.060‒1.257) when the coagulation disorder status was fixed. Furthermore, a patient with a coagulation disorder had 5 times more chance of dying when compared to an individual without the complication (OR = 5.15; 95% CI 1.279‒20.758) with the value of 24hOI fixed.

## Discussion

The present study sought to investigate mortality risk factors for CDH in a developing country reference center. Although no unique antenatal or post-natal index is able to define prognosis, risk stratification is an interesting approach in the sense that it enables better multidisciplinary care and family counseling. In the present study, the authors decided to evaluate exclusively post-natal risk factors, due to heterogeneity in the data registry and prenatal follow-up, a reality in our country, where pregnant women frequently have late referrals to tertiary centers and NB are sometimes diagnosed only after birth. This choice was reinforced by previously published data that shows that post-natal risk factors may be as useful for predicting survival and the need for ECMO as prenatal factors.^13^^,^^14^

Antenatal diagnosis allows delivery at reference centers with an appropriate level of care, which is paramount for improving survival results. Birth at a tertiary reference service enhances the chances of survival and is preferable to post-birth transportation.^15^ It is estimated that only 40%‒50% of NB with CDH are able to be admitted to a tertiary center and survival rates are proportional to rates of transference to these centers.^16^ Transference rates in our study were similar to previously published data, as were the rates of birth at a reference center. However, our study revealed a better survival rate for patients born outside of our hospital, at low-complexity centers (Table 4). This finding may be explained by the fact that the earlier the diagnosis is made antenatally, the more severe the malformation is, and/or the higher the chance for other congenital anomalies, which are risk factors for an earlier referral for these patients.^17^ Another finding that corroborates this theory is that patients who were born at our center had a higher chance for prematurity, low birth weight, cardiac anomalies and intrathoracic liver.

The predominance of the male gender has been previously described for this population,^18^ being considered a risk factor for death in previous studies. Although there is no absolute evidence that correlates gender with worse outcomes, the authors found a higher mortality rate for the female gender (Table 4). Additional risk factors were smaller birth weight and younger gestational age, a finding similar to previously published data.^19^

Among risk factors that can be identified both pre or postnatally, the intra-thoracic liver is a protagonist and has historically been associated with worse outcomes.^20^ This condition is implicated in higher rates of pulmonary hypoplasia and a more frequent need for patches in surgical repair.^21^ Our data showed that intra-thoracic liver, regardless of laterality, had an impact on mortality. Other risk factors that deserve attention are the combination with other congenital anomalies and genetic syndromes.^8^ Association between CDH and congenital heart disease has been shown to implicate lower survival rates, especially in cases of complex or univentricular heart diseases.^22^^,^^23^ The reason for this association is still unknown, and genetic, anatomic and blood flow-related features may play a role.^24^

Few studies have looked into the association between CDH and clinical complications. Levy et al.^25^ showed a 45% incidence rate of sepsis episodes, most frequently Ventilator-Associated Pneumonia and central line-associated bloodstream infections. Gestational age at birth and intra-thoracic liver were significantly associated with the occurrence of sepsis, and infected patients had longer ICU stay, as the authors also observed in the present study. The relationship between mortality and pneumothorax in CDH has been reported.^26^ Although the present data did not show statistical significance, the authors noted that 71% of patients who developed pneumothorax did not survive. Larger diaphragmatic defects, require of a prosthetic patch, worse 24hOI and elevated MAP could explain this association.^26^ The relationship between coagulation and CDH has not yet been studied; the authors observed that coagulation disorders were a feature in 2/3 of the patients, but it was a finding not significantly associated with mortality. The authors hypothesized that the association may be explained by limited reserves of pro- and anticoagulant factors, since these patients are often subjected to a recurrent scenario of systemic inflammation, in which hypoxia, lability of blood pressure levels, immaturity, and multiple invasions of skin and mucous membrane barriers are present, potentially precipitating factors of coagulopathy.^27^

Both the use of HFOV and vasoactive drugs were associated with higher mortality rates in the present series. Since there is a wide range of protocols and resources among centers, the choice between HFOV and CMV as the initial ventilation mode is still controversial, with HFOV being reserved for CMV failure at some centers,^28^ including ours. These patients may be classified as a group of greater severity, a possible reason for the noted higher mortality. Accordingly, the VICI-trial study showed that the HFOV group had a greater need for vasoactive support, iNO and ECMO and also initially presented with higher initial MAP when compared to the CMV group.^29^ The relationship between iNO and mortality was a finding in the present study, in resonance with previously published data.^28^ Since sicker babies require a wider therapeutic arsenal, this association may be explained by a severity bias.

The severity of PH is an important factor related to mortality in CDH. Newborns with CDH have to struggle with abnormal persistence of elevated pulmonary vascular resistance. Studies have aimed at establishing its clinical involvement based on echocardiographic parameters.^30^^,^^31^ In a retrospective analysis, Dillon et al.^32^ found evidence that the relationship between SPAP/SBP could subdivide patients into different outcomes, with suprasystemic SPAP being associated with no survival. The present data also showed the relevance of this relationship, with 100% survival in cases with SPAP < 2/3 SBP, and 66% mortality in cases with suprasystemic SPAP. Similarly, there has been an association between a worse evolution and the VTIp measurement, reflecting the PH and the right ventricle dynamics, as well as the higher graduation achieved in the ductal shunt scale.

The role of the left heart in circulatory collapse had its first investigation in the observation of small left cardiac chambers in patients with left CDH.^33^ In addition to the initial hypothesis that the presence of abdominal viscera could lead to compression of the cardiac mass and hypoplasia of its structures, another hypothesis that is also accepted is that anatomic distortion decreases the amount of blood flow across the foramen ovale leading to chronic underfilling of the left side of the heart and decreased LV growth also contributing to the observed underdevelopment.^34^ In the present study, reduction in both anatomical and functional LV measures were risk factors for death.

Prognostic indexes are an important tool to predict poor outcomes. The Apgar score, which is one of the first and most commonly known neonatal prognostic index, has been well-studied as a prognostic factor for CDH.^35^, ^36^, ^37^ SNAPPE-II^38^^,^^39^ and Oxygenation Index^37^^,^^40^ have also been used to assess the severity for this population. All three prognostic indexes were significantly associated with mortality in the present study's cohort, in accordance with previous studies. The oxygenation index was also relevant in the multivariate analysis and, as did lower birth weights, added increased potential to low survival rates.

## Conclusions

Prognostic indexes are an important tool for predicting outcomes and improving resource allocation. Post-natal risk factors may be more suitable for settings where antenatal diagnosis is not universal. Classical risk factors, such as prematurity, low birth weight, higher need for supportive care, and poorer prognostic indexes were associated with mortality in the CDH population. Risk stratification may help family counseling and improve the multidisciplinary approach for this spectral disease.

### Study limitation

The retrospective and single-center nature of the present study is an inherent limitation. This aspect limits the wider applicability of the present results on a large scale for patients with CDH; however, at the same time, it is interesting for being part of an existing context in emerging countries, where advanced infrastructure such as ECMO may not be readily obtainable.

The narrow sample may also play a role in the interpretation of other factors that have already been described as relevant for this population, such as prematurity, laterality of the hernia and the presence of intrathoracic liver.

## Funding

This research did not receive any specific grant from funding agencies in the public, commercial, or not-for-profit sectors.

## Declaration of Competing Interest

The authors declare no conflicts of interest.
